# Unlocking the Potential of Acetazolamide: A Literature Review of an Adjunctive Approach in Heart Failure Management

**DOI:** 10.3390/jcm13010288

**Published:** 2024-01-04

**Authors:** Michael Sabina, Zein Barakat, Adrian Feliciano, Andrew Lamb, M Mrhaf Alsamman

**Affiliations:** Lakeland Regional Health Medical Center, Lakeland 33805-4500, FL, USA; zein.barakat@mylrh.org (Z.B.); adrian.feliciano@mylrh.org (A.F.); andrew.lamb@mylrh.org (A.L.); mmrhaf.alsamman@mylrh.org (M.M.A.)

**Keywords:** acetazolamide, heart failure, diuresis, literature review, trials, natriuresis

## Abstract

**Background:** Heart failure (HF) patients often experience persistent fluid overload despite standard diuretic therapy. The adjunctive use of acetazolamide, a carbonic anhydrase inhibitor, in combination with loop diuretics has shown promise in improving decongestion and diuretic efficacy. This literature review aims to analyze six studies evaluating the effectiveness of acetazolamide as an additive treatment for acute decompensated heart failure (ADHF) and its impact on various outcomes. **Methods:** We searched the PubMed database using the terms “acetazolamide heart failure”. We refined our search with specific filters (as shown our PRISMA flow diagram) and exclusion criteria, narrowing down our results to five studies. We included an extra study via expert recommendation, ultimately including six studies for comprehensive analysis. **Results:** The review highlights the positive effects of acetazolamide on decongestion, natriuresis, and diuresis in HF patients. However, it also showcases the limitations of these trials. **Discussion:** While the reviewed studies demonstrate the potential benefits of acetazolamide in enhancing decongestion and diuretic efficiency, there are limitations to consider, including small sample sizes, lack of blinding, and limited external validity. Further research is needed to confirm these findings, compare acetazolamide with other diuretic combinations, and explore its effects in a broader population of heart failure patients, including those in the United States. The use of acetazolamide in HF management warrants continued investigation to optimize its role in improving decongestion and patient outcomes.

## 1. Introduction

Acute decompensated heart failure (HF) is a leading cause of hospitalizations in the United States. Per the American Heart Association 2022 guidelines for management of HF, hospitalizations in the U.S. saw an 26% rise from 2013 to 2017 [1]. The primary treatment for these patients is diuretic therapy, aimed at alleviating symptoms through decongestion. However, diuretic resistance is a common complication, challenging the management of this condition. Recent studies highlight the potential of combining loop diuretics with Acetazolamide, a carbonic anhydrase inhibitor, to address this resistance. Although FDA-approved for congestive heart failure, Acetazolamide’s role as an adjunct diuretic remains underexplored. This review analyzes six studies on the combined use of loop diuretics and Acetazolamide in acute decompensated heart failure (ADHF).

## 2. Methodology

**Figure 1:**
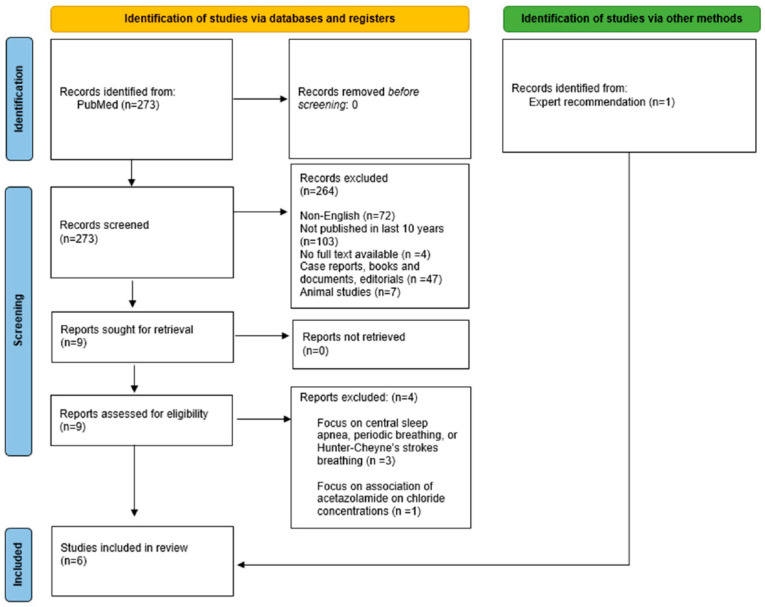
PRISMA Flow Diagram of Literature Search Methodology.

Our methodology, depicted in the PRISMA flow diagram (Figure 1), commenced with a search on the PubMed database using the terms “acetazolamide heart failure”. We refined our search with specific filters, as shown in the PRISMA flow diagram, which narrowed our results to nine studies. Of these, four were excluded due to their lack of relevance to the objectives of our literature review, ultimately including six studies for comprehensive analysis.

## 3. Characteristics of Acetazolamide

Acetazolamide, a diuretic with a spectrum of FDA-approved indications, is central to this review for its role in acute decompensated heart failure management. Acting as a carbonic anhydrase inhibitor, acetazolamide targets the enzyme within the nephron’s proximal tubule. This action impedes bicarbonate and, consequently, sodium reabsorption via interaction with the sodium hydrogen exchanger 3, enhancing their excretion and producing a diuretic effect via osmosis while also altering urine and blood pH. The diuresis produced is minimal when used in isolation, largely because reabsorption occurs further down the nephron tubule [2]. However, in adjunct with loop diuretics, it may potentiate its effects. This pharmacologic profile is particularly useful for correcting metabolic alkalosis in patients with heart failure who are also receiving loop diuretics, which can induce this condition [3]. This becomes particularly advantageous for acetazolamide when considering a study by Loon and Wilcox that showed alkalosis reduced the diuretic and natriuretic response of bumetanide [4].

**Figure 2:**
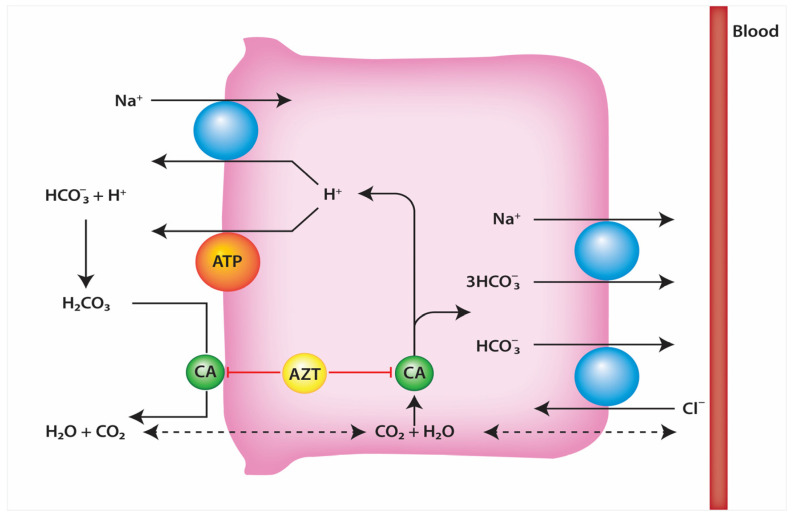
Acetazolamide’s Method of Action in the Nephron.

The drug’s unique influence on chloride ions in the serum distinguishes it from other diuretics as well; inhibition of carbonic anhydrase disrupts the bicarbonate-chloride exchange, leading to increased serum chloride levels. This becomes another vantage point for acetazolamide when evidence shown in a study by Hanberg et al. that hypochloremia was associated with diminished diuretic response is considered [5]. It is available in various dosages (125 mg, 250 mg, and 500 mg tablets) and forms, including intravenous preparations. Studies have demonstrated that IV acetazolamide is superior to the oral formulation in terms of reducing bicarbonate in 24 h, which is important to note when considering diuresis in cases of severe metabolic alkalosis [6]. Acetazolamide should be used with caution. It is contraindicated in severe renal disease and cirrhosis, and while its safety in pregnancy remains uncertain, it may be prescribed when the benefits justify the potential risks. In those with electrolyte abnormalities like hyperchloremia and metabolic acidosis, acetazolamide should be deferred (Figure 2).

## 4. Diuretic Resistance

The phenomenon of diuretic resistance encompasses a range of mechanisms. First, nephron remodeling is the ability of different segments of the nephron that are molecularly distinct to contribute to net sodium reabsorption. Loop diuretics primarily inhibit salt reabsorption along the thick ascending limb, but they do not increase sodium excretion elsewhere. In fact, they stimulate proximal and distal reabsorptive rates of sodium. This is one of the most common issues in patients who are chronically on loop diuretics. Second, the diuretic braking phenomenon refers to the progressively diminished effectiveness of a diuretic with each subsequent day of use when initiating treatment [7]. Another mechanism is post-diuretic sodium retention; when the nephron is exposed to an initial dose of a loop diuretic, the nephron responds to the extracellular volume initial loss by increasing reabsorption. This was evidenced in a study by Almeshari et al., where subjects received either a placebo or bumetanide with volume replacement (to counter sodium loss) in random order. Following bumetanide use, after being given NaCl, over the next 48 h subjects expelled 94% in the placebo group, and only 9% in the bumetanide group [8]. This study highlights the nephron’s ability to compensate for artificially induced losses. Lastly, reduced extracellular fluid volume causes reduced renal blood flow (RBF) and glomerular filtration rate (GFR), which can impair diuretic delivery to the kidneys [9]. The activation of RAAS in response to volume depletion will further exacerbate RBF and GFR reduction [10].

**Figure 3:**
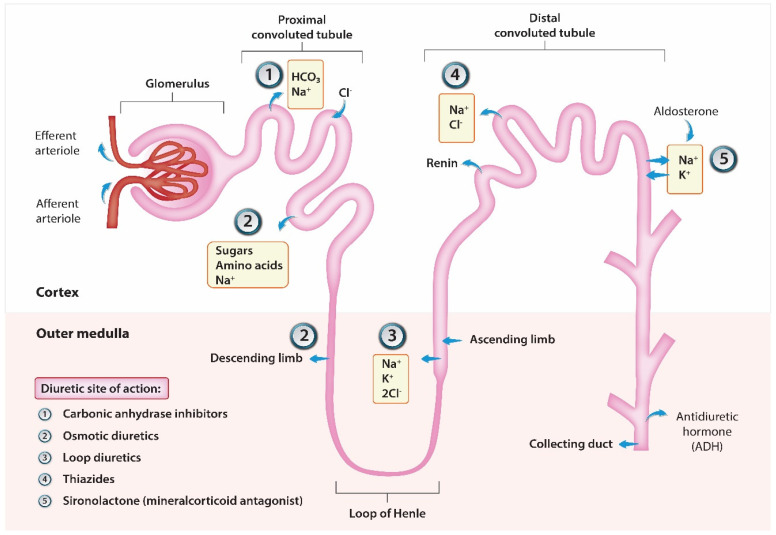
Diuretic sites of action.

Consequently, overcoming diuretic resistance remains a focal point of research. Combination therapy, targeting various nephron segments to amplify efficacy and overcome the body’s natural safeguards in maintaining homeostasis, holds significant promise. Researchers have been aggressive in the last decade in the quest for the best adjunct to loop diuretics when faced with acute decompensated heart failure and diuretic resistance. Figure 3 shows the diuretics of interest and where their sites of action in relation to loop diuretics are. Thiazide diuretics have been studied thoroughly as an adjunct to loop diuretics in multiple trials. The CLOROTIC trial tested hydrochlorothiazide with intravenous loop diuretics and showed improved diuretic response and weight loss compared with loop diuretics alone; however, there was no difference in mortality or rehospitalizations [11]. A study by Brisco-Bacik et al. examined metolazone as an adjunct to loop diuretics; mortality was increased and the diuretic effect attenuation was minimal [12]. Potassium sparring diuretics have also been covered as an adjunct in the ATHENA-HF trial, which showed no significant improvement in primary or secondary efficacy endpoints [13]. Vasopressin antagonists were tested in two separate studies known as ACTIV in CHF and TACTICS-HF [14,15]. The combination of the studies did reveal that there was improved weight loss, but there were no differences in clinical outcomes, mortality, or rehospitalizations.

Together, these trials highlight the recent effort by researchers to find a solution to diuretic resistance. This review will attempt to analyze six studies that test acetazolamide as an adjunct to loop diuretics.

## 5. Study Analysis

### 5.1. Acetazolamide as Add-On Diuretic Therapy in Exacerbations of Chronic Heart Failure: A Pilot Study by Imiela et al., 2017 [16]

The study in question is designed as a single-center, open-label pilot investigation conducted in a Polish hospital, focusing on 20 patients admitted due to acute exacerbations of heart failure with fluid overload. The average age of the participants was 72 years, with 85% being male. The study cohort comprised individuals with a left ventricular ejection fraction (LVEF) below 50%, with the mean LVEF measuring 33.9 ± 11.4%. Patients were randomized to receive oral Acetazolamide once daily, with dose adjustments based on body weight (<75 kg, 250 mg; 75–100 kg, 375 mg; >100 kg, 500 mg) versus a control group receiving only furosemide alone. To evaluate the impact of Acetazolamide on fluid output while patients were already on diuretics prior to acetazolamide initiation, Acetazolamide was administered solely on days 2 and 3. Over the course of four days, the study assessed diuresis, natriuresis, fluid balance, and symptoms.

The acetazolamide group did not exhibit significant differences in natriuresis and diuresis but did demonstrate a considerably more negative fluid balance when compared to the control group on days 3 and 4 (with a difference of −541 mL vs. +242 mL in fluid balance). Additionally, the acetazolamide group showed significantly reduced dyspnea scores on days 2–4, as measured by the visual dyspnea scale and Likert scale. These findings support the use of Acetazolamide as an adjunct to loop diuretics in promoting improved fluid balance and symptomatic presentation.

However, the study has several limitations that warrant consideration. First, the study lacks significant power, with a sample size of 20. The open-label study design may introduce reporting bias in measurements and dyspnea scores, as participants and healthcare providers were aware of treatment assignments. There is also the possibility that physicians may have adjusted background diuretics differently for patients receiving Acetazolamide. The broad standard deviations in average ejection fraction are noteworthy, as they could categorize patients differently based on modern guidelines (e.g., HFmrEF, HFpEF, HFrEF). The 2012 European Society of Cardiology (ESC) guidelines were used as a reference, categorizing LVEF into three ranges: normal EF (>50%), “grey-zone” EF (35–50%), and reduced EF (<35%). The study did not specify which heart failure (HF) categories exhibited responses to experimental treatment. The reliance on 2012 guidelines for heart failure management may not align with current practices and fails to highlight which patient population depending on severity of heart failure can benefit from this adjunctive therapy. Lastly, it is essential to note that Poland has different guidelines in management and healthcare for patients with heart failure; the study’s external validity is in question as to whether it can be generalized to patients in the United States.

In conclusion, while this study does highlight the potential of acetazolamide as adjunct therapy to promote better fluid balance and symptom control, its limitations necessitate caution in interpretation. Further investigation, ideally in the form of a more extensive, blinded trial with updated classification criteria, is required to confirm the observed positive effects of Acetazolamide.

### 5.2. Use of Acetazolamide in the Treatment of Patients with Refractory Congestive Heart Failure by Núñez et al. (2018) [17]

This was an observational retrospective study in Spain that evaluated the effects of oral acetazolamide as an add-on therapy in patients with refractory congestive heart failure (CHF). The study aimed to assess changes in functional class and indicators of fluid overload, as well as renal function, serum electrolytes, and pH after the addition of acetazolamide to standard intensive diuretic treatment. The 25 patients were selected based on specific criteria, including a diagnosis of heart failure, elevated plasma amino-terminal probrain natriuretic peptide (NT-proBNP) levels, NYHA functional class ≥ III, and persistent signs of fluid overload despite intensive diuretic treatment. The primary endpoint was to determine the clinical evolution of patients in terms of long-term changes in NYHA class and surrogates of fluid overload. Secondary endpoints included the follow-up trajectories for estimated glomerular filtration rate (eGFR), urea, serum sodium and potassium, pH, and systolic blood pressure (SBP). The study found that after the prescription of acetazolamide, there was a significant decrease in NYHA class, weight, and antigen carbohydrate 125, indicating an improvement in functional class and surrogates of fluid overload. Additionally, eGFR increased over time, suggesting an improvement in renal function. There were no significant changes in systolic blood pressure, serum sodium, potassium, NT-proBNP, and pH. The study also reported that the addition of acetazolamide did not lead to any alarming safety issues, such as detrimental effects on electrolytes and acid-base homeostasis.

The study showed the addition of oral acetazolamide to an intensive diuretic regimen in patients with refractory CHF is feasible, safe, and associated with an improvement in functional class and surrogates of fluid overload. The study acknowledges several limitations, including the small sample size, the retrospective and observational nature of the study, the lack of a control group, and the potential effects of other treatment changes during follow-up. Additionally, 68% of patients were receiving mineralocorticoid receptor antagonists (MRA), 88% were taking thiazides, and 56% were on triple diuretic therapy at baseline. This obscures the positive results of the study and it is unclear if acetazolamide is the reason for the results. Most of the patients were also on treatment with oral potassium supplements, 92% were confirmed, further obscuring the data on side effects and electrolyte imbalances. In our opinion, no decisions or recommendations should be taken into clinical practice from the results of this study alone.

### 5.3. DIURESIS-CHF TRIAL by Verbrugge et al., 2019 [18]

DIURESIS-CHF was a prospective, randomized trial conducted in Belgium from 2013 to 2017, enrolling 34 total patients with acute decompensated heart failure. Eligible patients were adults aged 18 years and older, displaying signs of volume overload (such as edema, ascites, jugular venous distention, and pulmonary congestion), with a left ventricular ejection fraction (LVEF) below 50%, and NT-proBNP levels exceeding 1000 ng/L. Additionally, patients needed to exhibit at least one risk factor for diuretic resistance, including hyponatremia, impaired renal function, or an elevated BUN/creatinine ratio. The study cohort was characterized a mean age of 80 years, advanced heart failure, with 43% having reduced ejection fraction, and a median NT-proBNP level of 7849 ng/L. Participants were randomly assigned to one of three groups; aetazolamide 250 mg daily in addition to low-dose loop diuretics (bumetanide 1 mg twice daily), acetazolamide 500 mg daily alongside high-dose loop diuretics (bumetanide 2 mg), and high-dose loop diuretics alone (twice the home maintenance dose) for 72 h.

The primary endpoint of the study was 24-h natriuresis. Secondary endpoints included reductions in NT-proBNP levels, worsening renal function (WRF), neurohormonal activation, and clinical outcomes. Surprisingly, 24-h natriuresis levels were found to be similar between the Acetazolamide plus low-dose bumetanide group (264 ± 126 mmol) and the high-dose bumetanide-alone group (234 ± 133 mmol, *p* = 0.515). However, loop diuretic efficiency, defined as natriuresis corrected for loop diuretic dose, was significantly higher with Acetazolamide (84 ± 46 vs. 52 ± 42 mmol/mg, *p* = 0.048). NT-proBNP levels decreased from 8165 ng/L to 6341 ng/L after 72 h in the acetazolamide group (*p* = 0.001) and from 7339 ng/L to 5746 ng/L in the loop diuretic-alone group. The two groups’ relative change in NT-proBNP levels was similar (−12 ± 38% vs. −9 ± 40%, *p* = 0.829). Worsening renal function was more prevalent in the acetazolamide group when compared to control group (defined as >0.3 mg/dL creatinine rise; 28% vs. 0%, *p* = 0.046). Additionally, as determined by peak plasma renin activity and aldosterone levels, neurohormonal activation did not differ significantly between the groups. Although there was no substantial difference in time to all-cause mortality, the time to mortality or heart failure readmission exhibited a non-significant numerical advantage for the acetazolamide group (*p* = 0.098).

In summary, the addition of acetazolamide as an adjunct to loop diuretics offers an effect of enhancement in loop diuretic efficiency through carbonic anhydrase inhibition. It should be noted that this effect also led to a higher incidence of “worsening renal function”, although the implications of this are subject to debate. On the other hand, this study has limitations worth considering. The sample size of 34 hurts the power of the study. The absence of blinding represents a potential source of bias. The generalizability of these findings to the United States, where heart failure management guidelines differ for outpatient and inpatient settings, remains to be determined. The study did not delineate the categorization of patients according to their ejection fraction, nor did it explain the rationale for the use of Acetazolamide with bumetanide as opposed to furosemide, the more conventional diuretic of choice in these scenarios.

### 5.4. ADVOR-Trial by Mullens et al., 2022 [19]

This was a multicenter, double-blind, placebo-controlled randomized trial across 27 medical sites in Belgium with 519 patients admitted with acute decompensated heart failure. Patients presented with at least one sign of fluid overload and an elevated natriuretic peptide (>1000 pg per mL or B-type natriuretic peptide level > 250 pg per mL). Patients were randomized to receive either an intravenous bolus of Acetazolamide 500 mg daily or placebo for three days, with both groups receiving an intravenous loop diuretic bolus at twice the oral maintenance dose, administered twice daily with a six-hour interval, for the subsequent two days.

The primary outcome measure was the achievement of successful decongestion within three days, defined as the absence of fluid overload signs without the need for escalated therapy. Secondary outcomes included death or heart failure rehospitalization at three months and hospital length of stay. Successful decongestion within three days was achieved in 42.2% of patients receiving Acetazolamide compared to 30.5% in the placebo group (*p* < 0.001). The acetazolamide group also exhibited a more significant reduction in clinical congestion scores, with 79% of patients demonstrating successful decongestion upon discharge, compared to 63% in the placebo group. Acetazolamide was associated with a reduction in hospital stay by an average of 1.1 days (95% CI, 9.1 to 10.8). However, there was no statistically significant difference in the occurrence of death or heart failure rehospitalization at three months (29.7% vs. 27.8%) between the two groups. Crucially, the study found that Acetazolamide’s benefits were consistent across the spectrum of left ventricular ejection fraction (LVEF), irrespective of whether patients had heart failure with reduced ejection fraction (HFrEF), heart failure with mildly reduced ejection fraction (HFmrEF), or heart failure with preserved ejection fraction (HFpEF). This suggests that Acetazolamide can be efficacious across various heart failure subtypes.

In conclusion, this meticulously designed, placebo-controlled, randomized trial provides compelling evidence that adding acetazolamide to intravenous loop diuretics leads to more effective decongestion in patients hospitalized for acute heart failure with fluid overload. The ADVOR trial is the most robust regarding acetazolamide and its adjunct potential to loop diuretics in treating acutely decompensated heart failure, thus putting it at the forefront for critique. There have been several meta-analyses and post hoc analyses of this trial with interesting findings. Meekers et al. found that acetazolamide was associated with a higher rate of successful decongestion across the entire range of renal function, with more pronounced effects regarding natriuresis and diuresis in patients with a lower eGFR. While WRF occurred more frequently with acetazolamide, this was not associated with adverse clinical outcomes [20]. In a critical editorial by Bueno and Packer, acetazolamide showed modest increases in urinary sodium excretion and volume but not enough to exhibit substantial diuresis as expected for true decongestion [21]. In patients with an eGFR > 40 mL/min/1.73 m^2^, acetazolamide’s impact on urinary sodium and volume was minimal and not statistically significant. Nonetheless, successful decongestion is reported, raising queries about how this was achieved without significant natriuresis or diuresis. Another interesting critique by Bueno and Packer noted that a physical assessment of congestion was blinded. However, physicians might have been influenced by changes in serum bicarbonate levels, a well-known effect of acetazolamide, thus creating bias and attributing successful congestion due to changes in bicarbonate levels rather than actual decongestive effects of the drug. Using a non-validated congestion score without assessing inter-rater reliability was a known limitation by the researchers of the ADVOR trial.

### 5.5. CANDI by Martin et al. (2022) [22]

The CANDI study is a retrospective observational study involving 55 patients with acute heart failure who demonstrated poor diuretic response. It is the first of its kind in comparing acetazolamide head-to-head with another diuretic as an adjunct therapy to loop diuretics in ADHF. The study evaluated the initial effects and safety of adding either acetazolamide (median dose: 125 mg, range 125–250 mg) or chlorthalidone (median dose: 25 mg) to furosemide (range: 120–180 mg) on day 7 after being on standard diuretic therapy. The primary objective was to observe the proportion of patients achieving at least 1 kg of weight loss within 24 h post-administration of the second diuretic. Other outcomes analyzed were diuresis, natriuresis, and variations in renal function and electrolytes.

After adjusting for various factors, the effect of acetazolamide compared to chlorthalidone was significant, with a greater proportion of patients in the acetazolamide group achieving the desired weight reduction. Results indicated the treatment effect was 0.36 in favor acetazolamide over chlorthalidone (CI 95%: 0.09–0.63, *p* = 0.008). Futhermore, 62% of participants demonstrated a reduction of at least 1 kg in weight following the addition of acetazolamide when compared to the 26% of chlorthalidone. No significant differences were observed in diuresis and natriuresis between the two groups. Changes in pH and bicarbonate levels were significant, obviously showing acetazolamide lowered pH, a known effect. Changes in renal function were not statistically significant between groups.

These are positive findings for acetazolamide as it is data that reinforces its use over a thiazide diuretic when facing diuretic resistance, assuming there are no obvious contraindications to either drug. It appears superior at preventing metabolic alkalosis, and there is no difference in worsening renal function or causing electrolyte disturbances. The study, however, has limitations. Its sample size is small, limiting its power. Due to its observational nature, it can only suggest associations, not causative relationships, between treatments and outcomes.

### 5.6. Diuretic, Natriuretic, and Chloride-Regaining Effects of Oral Acetazolamide as an Add-On Therapy for Acute Heart Failure with Volume Overload by Kosiorek et al., 2023 [23]

This prospective, randomized study in Poland is the latest trial investigating the effects of acetazolamide as an additional treatment for patients with acute heart failure (AHF) experiencing volume overload. It aims to assess the decongestive, natriuretic, and chloride-regaining effects, as well as the renal safety profile, of oral acetazolamide (250 mg) used alongside standard care in these patients. The study’s outcomes include measurements of diuresis, fluid balance, weight loss, natriuresis, serum chloride concentration, serum creatinine concentration, estimated glomerular filtration rate (eGFR), occurrence of worsening renal function (WRF), and urinary levels of kidney biomarkers (neutrophil gelatinase-associated lipocalin [NGAL], kidney injury molecule-1 [KIM-1], and cystatin C [Cys-C]).

The results show that the acetazolamide group demonstrated significantly more fluid loss (diuresis) in all three days and weight loss after the 48 and 72 h when compared to those who did not receive the drug. Natriuresis was significantly higher on days 2 and 3, as measured by urinary sodium levels. Serum chloride concentration, compared to controls, was significantly higher on days 2 and 3, with no increase in creatinine concentration and urinary renal biomarker levels. This trial highlights acetazolamide’s positive diuretic and natriuretic effects as an adjunct therapy, and goes further to showcase its chloride regaining effects and renal safety.

The study comes with its limitations, most notably in the form of its lack of power due to a relatively small sample size (61). Additionally, the study did not thoroughly assess the impact on heart failure symptoms, such as edema and rales, and the measurement of specific renal biomarkers, while insightful, might not be practical in everyday clinical settings.

## 6. Discussion

### 6.1. Summary of Trial Analysis

We have created a table (Table S1) that summarizes all six studies in a brief manner.

In summary, there are positive findings, but several limitations are apparent. Most notably, small sample sizes hurt the power of these studies. The absence of double-blind designs brings up concerns about potential bias. Assessments of successful decongestion with non-blinded studies or non-validated scoring for decongestion is questionable. Lastly, the exclusive focus on patient populations in Europe, or more specifically, Belgium and Poland, may limit the generalizability of findings to a broader, more diverse patient population. There is also a concern of adherence to current guidelines in regard to heart failure classifications and treatment protocols.

However, these trials show significant promise in regard to acetazolamide as an adjunct for the treatment of ADHF. A meta-analysis by Malik et al. in 2023 wraps up the trials clearly and shows that successful decongestion was significantly higher in patients receiving Acetazolamide compared to the control group. Mean natriuresis and diuresis was significantly higher in acetazolamide patients. Consistent with all individual studies, no significant difference was found between the two groups in terms of all-cause mortality and hospitalization due to heart failure [24].

### 6.2. Acetazolamide Standing Amongst Other Adjuncts

In addressing diuretic resistance in heart failure management, the critical question is not the efficacy of adjunct therapy to standard loop diuretics, but rather the specific benefits of acetazolamide and its comparative effectiveness against other adjunctive diuretics. Acetazolamide’s established safety and beneficial role seem well established. However, comparative efficacy necessitates further research, particularly through head-to-head trials with alternatives. Although the CANDI trial, comparing chlorthalidone with acetazolamide, had limitations in its observational, retrospective design and lacked statistical power, it provided insightful findings, demonstrating acetazolamide superiority in promoting weight loss, reducing serum pH, and counteracting metabolic alkalosis [22]. Additionally, concerns regarding its potential to worsen renal function were not apparent when compared with a thiazide diuretic.

Future research might benefit from exploring whether adjunctive proximal diuresis, as facilitated by agents like acetazolamide or SGLT2 inhibitors, offers superior outcomes in heart failure management compared to adjunctive distal diuresis, as facilitated by agents like thiazide diuretics, potassium sparring diuretics, and vasopressin antagonists. This inquiry could examine the hypothesis that the nephron’s compensatory mechanisms in response to reduced reabsorption in the Loop of Henle might be more pronounced in proximal tubule, thus inhibiting that region might provide better diuretic efficacy.

A pertinent area of investigation would be a comparison between the most effective proximal tubule diuretics and the best distal tubule diuretics as adjuncts to loop diuretics. Additional research efforts might explore if chlorthalidone is the optimal diuretic adjunct distal to the Loop of Henle. In patients receiving IV loop diuretics for ADHF, chlorthalidone administration was associated with a greater short-term natriuresis in comparison to spironolactone in a study by Llacer et al. [25]. There are no studies, per our literature search, comparing thiazides with vasopressin antagonists.

When considering the similar osmotic diuresis effects of acetazolamide and SGLT2 inhibitors in the proximal tubule, it is well known SGLT2 inhibitors display benefits in the management of heart failure in long term management. There are no studies that show acetazolamide’s benefits long term in the management of heart failure. However, it is also increasingly becoming evident acetazolamide has short term benefits in the treatment of acutely decompensated heart failure. On the other hand, empagliflozin (SGLT2 inhibitor) does not report short term effects on diuresis or changes in physical signs of congestion as evidenced by the EMPULSE trial [26]. The EMPAG-HF and EMPA-RSPONSE-AHF trials further highlighted empagliflozin had no effect on diuresis and physical signs of congestion in the first four days [27,28]. A future study comparing SGLT-2 inhibitors with acetazolamide in a head-to-head trial that compares short-term and long-term outcomes would shed light possibly on why two diuretics with similar mechanisms have benefits in different intervals of heart failure.

However, the additional benefits of acetazolamide in addressing metabolic alkalosis and correcting chloride imbalances suggest its unique therapeutic role. Research by Kartaoka (2019) and Zandijk (2021) on the chloride theory emphasizes the importance of chloride management in diuretic response [29,30]. Acetazolamide’s unique impact on serum chloride levels and its role in addressing hypochloremia may offer an advantage over other diuretics.

### 6.3. Recommendations for Acetazolamide as Adjunct Therapy

As it stands, acetazolamide appears to be a safe and effective adjunct therapy in heart failure, especially in cases of metabolic alkalosis and chloride imbalance. The observation of worsening renal function in multiple studies raises concerns. While this does not appear to significantly alter outcomes like mortality and heart failure hospitalization, it does necessitate cautious monitoring of renal function during acetazolamide therapy. Until further research provides more definitive comparisons with other diuretics, acetazolamide should be considered a valuable option in specific scenarios of heart failure management, particularly where rapid decongestion is necessary, and in patients experiencing metabolic complications such as alkalosis or hyperchloremia. Preferably, in the formulation intravenously over oral tablets in ADHF settings.

**Figure 4:**
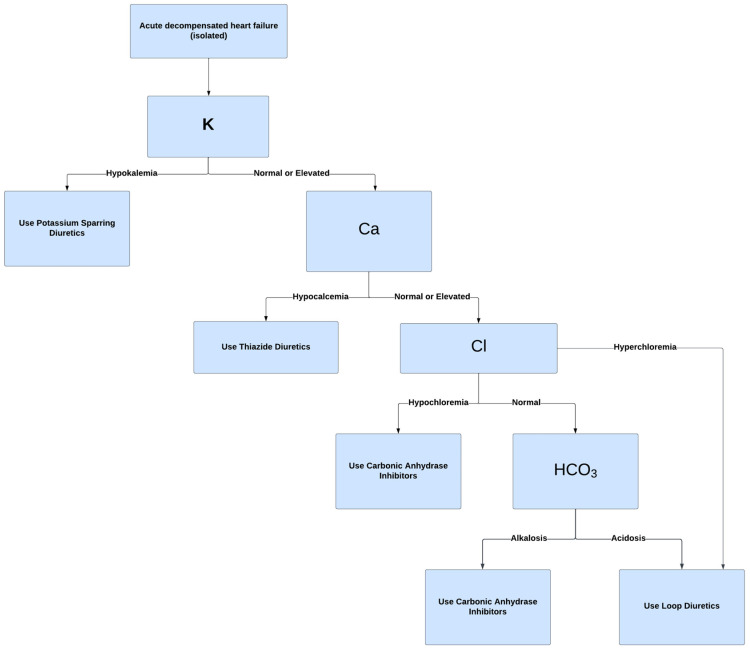
Diuretic Use Algorithm.

We propose a basic algorithm (Figure 4) to aid in deciding when to use acetazolamide for diuresis in heart failure, considering serum electrolytes. Of note, the algorithm’s utility is limited by the complexity of concurrent electrolyte imbalances often encountered clinically. In such cases, electrolyte supplementation may be required alongside diuretic therapy. Despite the potential utility of acetazolamide, loop diuretics largely remain the cornerstone of initial treatment for heart failure, with acetazolamide typically considered in more complex scenarios involving severe electrolyte disturbances.

## 7. Conclusions

Ultimately, while the current evidence highlights the potential benefits of Acetazolamide as adjunctive therapy with loop diuretics, head-to-head trials, and further well-designed, adequately powered trials are needed to solidify its place in the therapeutic armamentarium for heart failure and assess its impact on clinical outcomes.

## Data Availability

Data are contained within the article and supplementary materials.

## Supplementary Materials

The following supporting information can be downloaded at: https://www.mdpi.com/article/10.3390/jcm13010288/s1, Table S1: Acetazolamide Trial Table.
