# Supplementary material for: Unlocking the Potential of Acetazolamide: A Literature Review of an Adjunctive Approach in Heart Failure Management
Source: J Clin Med. 2024 Jan 4;13(1):288. doi: 10.3390/jcm13010288 (PMC10780103; doi:10.3390/jcm13010288)
Supplement: Supplementary file 1 [file jcm-13-00288-s001.zip › jcm-2780568-supplementary.pdf]

Study	Sample Size	Intevention(s)	Primary Outcomes Measured	Secondary Outcomes Measured	Primary Outcome Results	Secondary Outcome Results
**Imiela (2017) [16]**	20	Oral Acetazolamide daily dose adjustments based on body weight (<75 kg, 250 mg; 75 - 100 kg, 375 mg; >100 kg, 500 mg)	Mean diuresis in ml days 3 & 4 and days 2, 3, and 4.<br><br>Natriuresis in mmol/L on each day (1-4).<br><br>Fluid Balance in ml days 3 & 4 and days 2, 3, and 4.	Dyspnea measured on days 1-4	Day 3, 4 (ml): 2769.5 (P=0.41)<br>Day 2, 3, 4 (ml): 2603.6 (P=0.91)<br><br>Day 1-4 (mmol/L): 207.9, 212.9, 253.1, 258 (P= 0.4, 0.55, 0.81, 0.43)<br><br>Day 3, 4 (ml): -541 (P=0.025)<br>Day 2, 3, 4 (ml): -304 (P=0.18)	Visual Dyspnea Scale (mean ± SD)<br>Day 1: 26 (±27) P=0.12<br>Day 2: 18 (±18) P=0.14<br>Day 3: 16 (±21) P=0.038<br>Day 4: 9 (±9) P=0.0006<br><br>Likert Scale (mean ± SD):<br>Day 1: 2.278 (±1.1) P=0.13<br>Day 2: 1.722 (±0.7) P=0.0076<br>Day 3: 1.667 (±0.97) P=0.07<br>Day 4: 1.444 (±0.64) P=0.041
		Furosemide			Day 3, 4 (ml): 2434.50 Day 2, 3, 4 (ml): 2557.00<br><br>Day 1-4 (mmol/L): 260.88, 247.11, 240.7, 213.3<br><br>Day 3,4 (ml): 242, 95.3	Visual Dyspnea Scale (mean ± SD)<br>Day 1: 42 (±13)<br>Day 2: 41 (±16)<br>Day 3: 38 (±19)<br>Day 4: 35 (±16)<br><br>Likert Scale (mean ± SD)<br>Day 1: 3 (±0.8)<br>Day 2: 2.833 (±0.8)<br>Day 3: 2.778 (±1.4)<br>Day 4: 2.222 (±0.83)
**Nunez (2018) [17]**	25	Acetazolamide + Loop Diuretic 120mg/day	NYHA class<br>Weight<br>CA125<br>NT-proBNP	Estimated glomerular filtration rate (eGFR), urea, serum sodium, potassium, pH, and systolic blood pressure	Improvement in NYHA class (P=0.0018)<br>Reduction in weight (P = 0.023)<br>Decrease in CA125 (P=0.001)<br>NT-proBNP remained stable	GFR improved (P=0.002)<br>no significant changes for other outcomes
**DIURESE-CHF (2019) [18]**	34	Acetazolamide 250 mg daily + low-dose loop diuretics (bumetanide 1 mg twice daily)<br><br>Or<br><br>Acetazolamide 500 mg daily + high-dose loop diuretics (bumetanide 2 mg)	24-hour natriuresis (corrected for loop diuretic dose)	NT-proBNP levels<br>Worsening renal function (WRF)<br>Neurohormonal activation<br>Clinical outcomes	84±46 mmol<br>(P=0.048)	NT-proBNP: 8165 ng/L to 6341 ng/L<br>(P = 0.001)<br><br>WRF: 5 (28%)<br>(P=0.046)<br><br>Peak Plasma Renin Activity: 4.8 μg/L/h<br>(P=0.904)<br>Peak Plasma Aldosterone Acvitiy: 260 ng/L<br>(P = 0.756)<br><br>No statistical difference in clinical outcomes
		High-dose loop diuretics alone (twice the home maintenance dose)			52±42 mmol	NT-proBNP: 7339 ng/L to 5746 ng/L<br><br>WRF: 0 (0%)<br><br>Peak Plasma Renin Activity: 4.9 μg/L/h<br>Peak Plasma Aldosterone Acvitiy: 212 ng/L<br><br>No statistical difference in clinical outcomes
**ADVOR (2022) [19]**	519	Acetazolamide 500 mg IV once daily + Loop diuretic IV (2x oral maintenance dose)	Succesful Decongestion	Duration of stay<br>OR<br>All cause death or Rehospitalization for heart failure	42.2% (P<0.001)	8.8 Days (CI; 0.81–0.98)<br>76 Particpants (29.7%) Hazard ratio, 1.07 (0.78–1.48)
		Placebo + Loop diuretic IV (2x oral maintenance dose)			30.50%	9.9 Days<br>72 participants (27.8%)
**CANDI (2022) [22]**	55	Acetazolamide 125-250 mg + furosemide 120-180 mg	Diuresis (ml)<br>Natriuresis (mEq/L)<br>Weight loss (kg)		Diuresis: No significant difference (+2025 ml, 95% CI: -1551 to 5601; p = 0.260)<br>Natriuresis: No significant difference (+1311 mEq/l, 95% CI: -235 to 2859; p = 0.095)<br>Weight loss reduction of ≥1 kg: 62% (CI: 95%, 39-84)	
		Chlorthalidone 25 mg + furosemide 120-180 mg			Weight loss reduction of ≥1 kg: 26% (CI: 95% 10-41)	
**Kosiorek (2023) [23]**	61	Oral Acetazolamide 250mg + Lasix	Diuresis<br>Fluid balance (ml)<br>Weight loss (kg)		Diuresis: 24, 48, 72 hours; 2750 ml, (P = 0.05), 5300 ml (P = 0.01), 7800 ml, (P = 0.004).<br>Fluid Balance: Day 1-3; –1682 ml (P = 0.015), –1232 ml (P = 0.042), –1126 ml (P =	
		Lasix (standard of care)			Diuresis; 24, 48, 72 hours; 1850 ml, 3750 ml, 5500 ml	
